# Tissue Engineered Cartilage Repair Using Small-Incision Implantation of Decalcified Corticocancellous Bone Scaffold

**DOI:** 10.1016/j.eats.2024.103346

**Published:** 2024-12-10

**Authors:** Zhenlong Liu, Zhenchen Hou, Tong Pan, Weixin Ye, Chang Liu, Yingfang Ao, Xi Gong

**Affiliations:** a Department of Sports Medicine, Peking University Third Hospital, Institute of Sports Medicine of Peking University, Beijing, China; b Beijing Key Laboratory of Sports Injuries, Beijing, China; c Engineering Research Center of Sports Trauma Treatment Technology and Devices, Ministry of Education, Beijing, China

## Abstract

Treatment of cartilage injuries is a prominent topic internationally. Recently, tissue engineering has been used to repair cartilage injury. In China, several scaffolds have been approved for clinical trials aimed at cartilage repairing. The successful implantation of scaffold in cartilage defect areas is crucial for effective treatment. The procedure is crucial in ensuring that the scaffold is implanted successfully, which is fundamental for a good prognosis. International reports on tissue-engineered scaffold implantation technologies highlight both advantages and disadvantages. Because of limitations in the required equipment and materials, some of the surgical techniques used internationally are not applicable in China. To optimize the technology of tissue-engineered scaffolds for cartilage repair in China, this Technical Note discusses the implantation techniques and skills involved, focusing on small-incision implantation of decalcified cortex-cancellous bone scaffolds. This work contributes to standardizing the surgical procedures for tissue engineering scaffold technology in repairing cartilage injuries.

Articular cartilage is a crucial structure of knee joints. It is smooth and elastic, effectively reducing friction between bones and absorbing shock, playing a key role in maintaining joint function.^1^ As the result of its dense extracellular matrix and lack of vascular tissue, blood supply to articular cartilage is deficient. As a result, nutrition can only be derived from the synovial fluid, making it difficult for cartilage to regenerate and repair once injured.^2^ Such injuries may lead to osteoarthritis, causing functional degradation or even loss of function.^3^^,^^4^

Treatments for articular cartilage include joint debridement, microfracture surgery, and autologous chondrocyte transplantation.^5^, ^6^, ^7^, ^8^ Each method has its own advantages and disadvantages. Joint debridement and microfracture can relieve symptoms and are relatively easy to perform. However, they do not form hyaline cartilage at the injury site, and their long-term efficacy is limited. Autologous cartilage transplantation risks donor-site morbidity, whereas allogeneic transplantation faces supply limitations. Currently, microfracture remains the primary technique for cartilage repair. However, it remains a challenge that regenerated cartilage forms as fibrocartilage, lacking hyaline cartilage's biomechanical properties.

In recent years, autologous matrix-induced chondrogenesis (AMIC) technology has progressed significantly^1^^,^^9^, ^10^, ^11^ and proven to provide significant improvement in patients with symptomatic, large chondral defects.^12^ The AMIC technique involves adding a biomaterial covering after microfracture. This approach provides initial strength and mechanical protection for the blood clot−containing bone marrow mesenchymal stem cells^13^ while also serving as a 3-dimensional framework for stem cells to attach, proliferate, and differentiate. This technique enhances the biomechanical properties of regenerated cartilage, making it more similar to hyaline cartilage.^14^ The advantages of the AMIC technique include combining with microfracture surgery to complete the repair in a single operation and avoiding the need to harvest the patient's healthy autologous tissue, thus preventing secondary damage at the donor site. The scaffold's mechanical and differentiation-inducing effects result in regenerated cartilage that is superior to fibrocartilage and more similar to hyaline cartilage.^15^ This Technical Note will elaborate on the surgical techniques for arthroscopic and small-incision implantation of decalcified cortex-cancellous bone scaffolds to repair cartilage injuries.

## Surgical Technique

After conducting research and screening, we found that the biomechanical properties of decalcified cortical-cancellous bone closely resemble those of cartilage. The scaffold offers unparalleled advantages over synthetic materials in terms of both morphology and biomechanics. First, it has an elastic modulus and compressive strength comparable with cartilage. The scaffold consists primarily of natural collagen I, making it an ideal substrate for cell adhesion and growth. Second, it is biodegradable and can be readily absorbed by the body as a biological material. The scaffold also features a natural porous structure that facilitates cell ingrowth and interaction with cytokines and bioactive substances. The cancellous bone component provides a 3-dimensional porous structure for cell growth, whereas the cortical bone component delivers essential mechanical strength.^16^^,^^17^

### Preparation

#### Indications and Patient Evaluation

This technique is suitable for patients experiencing knee cartilage lesions ranging from 1 to 8 square centimeters in size who experience pain or diminished knee function affecting their daily activities and athletic pursuits. The recommended patient age bracket is 18 to 55 years. Exclusion criteria include the presence of rheumatoid arthritis, severe osteoarthritis, or significant joint malalignment.

#### Preoperative Planning

Preoperative magnetic resonance imaging is necessary to evaluate the severity of the cartilage and subchondral bone defects. Make sure that the patient has no evidence of rheumatoid arthritis, severe osteoarthritis, or significant joint malalignment. The method of anesthesia is spinal anesthesia. Required materials and equipment are decalcified cortical-cancellous bone scaffolds (Made by Peking University Third Hospital; Beijing, China), cartilage nails (Biofix; Bioscience Ltd., Tampere, Finland), decalcified cortical-cancellous bone scaffold positioning guide (Made by Peking University Third Hospital; Beijing, China), microfracture device, planer, scraper, grinding drill, as well as arthroscopy equipment.

### Surgical Procedure

#### Procedure for arthroscope and scaffold preparation

Once anesthesia is administered, perform microfracture after the debridement of the cartilage injury area via arthroscopy using a conventional portal. Establish the anteromedial (operation portal) and anterolateral (observation portal) portals to the knee joint. Explore and probe the defect area under arthroscopy. Clean the injured cartilage with a planer. Clean the unstable cartilage at the edge of the injured area with a scraper. Use the microfracture device to perform the microfracture. For special conditions such as hyperosteogeny, use a grinding drill to remove excess (hyperostosis) bone to create a smooth surface.

Remove the arthroscope and expose the site of injury by a small incision. On the basis of the defect's size and location, create a minimally invasive incision of approximately 3 cm near the defect. Gradually incise the skin and subcutaneous tissue. Further cleaning of the injured cartilage with a scraper is required to stabilize the edge of the cartilage. After making a small incision, accurately measure the size and shape of the defect under direct vision.

Select and trim the decalcified bone scaffold under direct vision. Select a decalcified cortical-cancellous bone scaffold of the appropriate specification and rehydrate it with normal saline. Trim the scaffold according to the measured size, first trimming the cortical bone scaffold, then adjusting the cancellous bone scaffold to match the depth of the cartilage defect. Avoid repeated handling of the scaffold's junction area while trimming to prevent cracks, which could compromise the scaffold's strength.

#### Procedure for Implanting Decalcified Cortical-Cancellous Bone Scaffolds via a Small Incision

Place the scaffold into the joint, ensuring the cortical bone is oriented toward the subchondral bone. Fix the decalcified bone scaffold in the defect area using the self-developed honeycomb positioning guide. Through the channels on the fixator's surface, use a 1.5-mm drill bit to drill through the cancellous and cortical bones, creating a bone tunnel 15 to 20 mm deep. On the basis of the tunnel's depth and location, select appropriately sized cartilage nails for implantation. Typically, 1.5 mm × 15 mm or 1.5 mm × 20 mm cartilage nails are used for fixation. Depending on the defect size, implant 2-4 cartilage nails to secure the scaffold. When inserting the cartilage nail, the knocking force should be gentle instead of violent, otherwise the cartilage nail may penetrates the cortical bone framework, resulting in fixation failure.

**Fig 1:**
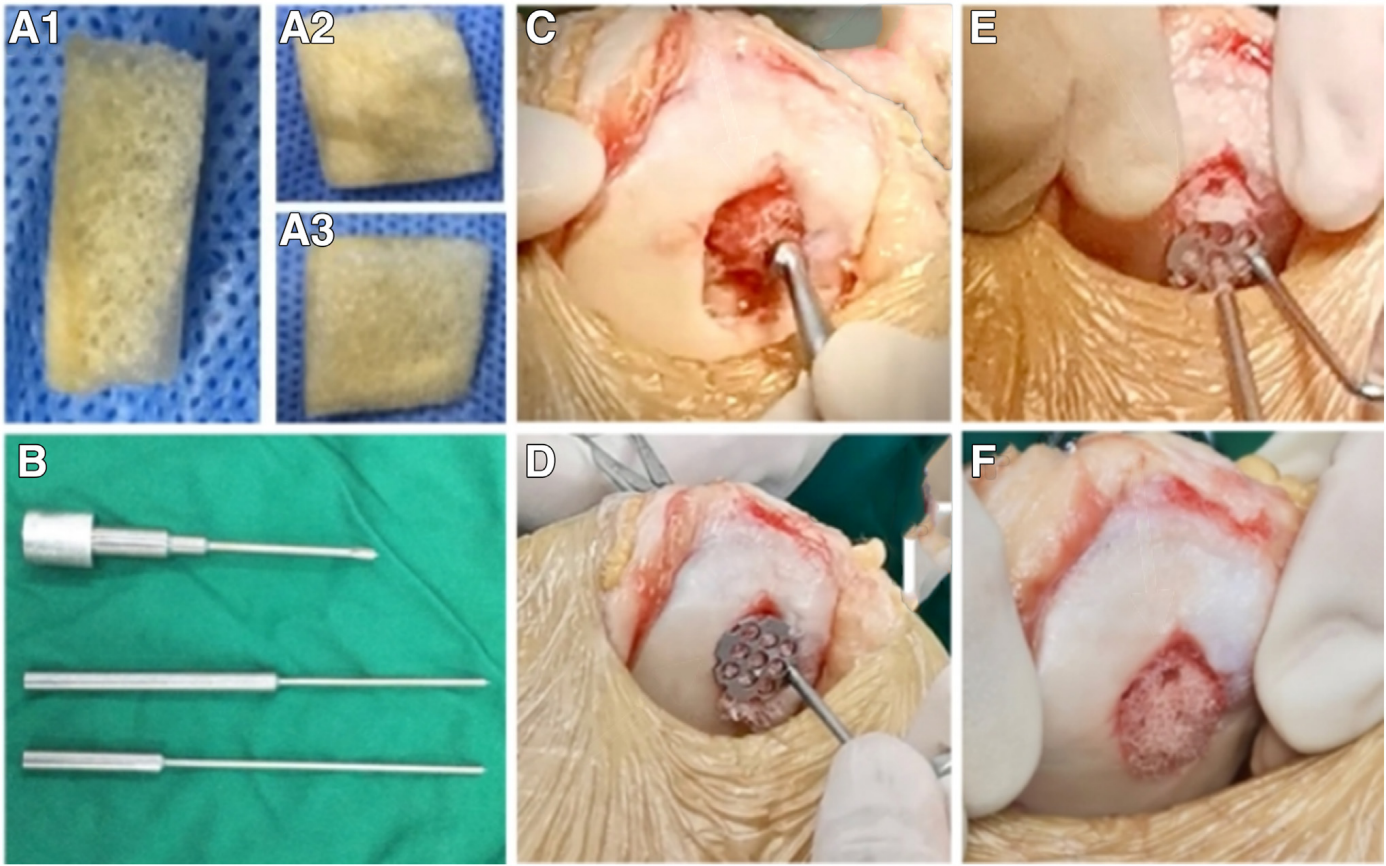
Materials and procedures for repairing patellar cartilage injury with a decalcified bone scaffold via small incision. (A1) Lateral view of the decalcified cortical-cancellous bone scaffold. (A2) View of the cortical bone surface of the decalcified cortical-cancellous bone scaffold. (A3) View of the cancellous bone surface of the decalcified cortical-cancellous bone scaffold. (B) The cartilage-positioning guide invented by the author's team, including the locator sleeve, depth-limiting positioning rod, and unlimited depth-limiting positioning rod, from top to bottom. (C) Direct view of the patient’s left knee with the patient in the supine position. Remove the arthroscope, then clean the injured cartilage further with a scraper under a small lateral patellar incision with a length of 5 cm to stabilize the edge of the cartilage. (D) Direct view of the patient’s left knee with the patient in the supine position. Through a small lateral patellar incision with a length of 5 cm, fix the scaffold on the lesion surface with the cortical bone facing the defect surface using the self-developed honeycomb demineralized bone scaffold positioning guide. (E) Direct view of the patient’s left knee with the patient in the supine position. A small lateral patellar incision with a length of 5 cm is made. Drilling of holes with a 1.5-mm electric drill and implantation of cartilage nails using the self-developed cartilage nail positioning guide. Depending on the defect size, 2-4 cartilage nails were implanted. (F) Gross view after completion of scaffold repair. Direct viewing of the patient’s left knee with the patient in the supine position. After fixing the scaffold, the raised part of the scaffold surface was cleaned with a planer to make it flush with the cartilage surface.

After securing the scaffold in place, trim its surface with a planer to ensure it is flush with the defect area. Flex and extend the knee joint repeatedly, 10 to 20 times, to check the stability of the fixation (Fig 1). Complete the surgery after thoroughly flushing the area, then immobilize the affected knee in an extended position using a pressure bandage and brace.

## Discussion

Cartilage injury is prevalent in clinical practice, often resulting from sports injuries, fractures, and aging, leading to structural and functional changes.^18^ Common methods used clinically have limitations. Microfracture can only generate fibrocartilage, which has poor long-term outcomes. Osteochondral transplantation is limited because of the high requirement for donors and lack of sources. Autologous chondrocyte implantation can cause secondary surgical trauma to patients. Tissue-engineered articular cartilage requires improvement. Most previous tissue-engineering scaffold operations require suturing, which prolongs the procedure and significantly affects patient recovery.

The surgical technique developed by the author's team offers unique advantages. First, we use a decalcified cortical-cancellous bone scaffold with independent intellectual property rights, demonstrating excellent biomechanical and biological characteristics. The scaffold's support layer ensures mechanical strength, whereas the functional layer enriches stem cells, accommodating more bone marrow−derived stem cells. This design accelerates stem cell proliferation and differentiation, effectively repairing cartilage defects. Second, the surgical technique's applicability is broader. Commonly used scaffolds in clinical settings have limitations for cases with large subchondral bone defects (>3 mm) since they are 2-dimensional structures, whereas our scaffold is more suitable because of its 3-dimensional structure. In addition, compared with traditional surgical suturing, our technique uses directly absorbable nail for stent implantation,^19^ which is simpler to operate and the fixation of the stent is more firm.

Despite these advantages, this technology has some shortcomings. The scaffold requires precise character matching and must align with the cartilage injury's morphology after manual trimming. An accurate measurement is essential for optimal implantation. In addition, the current surgical techniques still require a small incision, which is somewhat invasive for patients. Considering the current technology, future efforts should focus on completing the operation under arthroscopy to benefit more patients. Table 1 lists the advantages/pearls and disadvantages/pitfalls, including risks and limitations, of the surgical technique.

**Table 1:** Advantages/Pearls and Disadvantages/Pitfalls, Including Risks and Limitations, of the Surgical Technique

Advantages and Pearls	Disadvantages and Pitfalls
Features a 3-dimensional structure, which is especially suitable for patients with deep subchondral bone defects	The scaffold possess a 3-dimensional structure, which requires a small incision during implantation instead of arthroscopy
Human-derived, with good biocompatibility and low rejection reaction after decalcification	The scaffold's flexibility requires careful handling to avoid collapse during use.
The scaffold has a bilayer structure that fosters attachment on the cortical bone side and has inherent pores on the cancellous bone side, maintaining type Ⅰ collagen structure and enhancing MSC adhesion and proliferation.	The fixation technique demands precision. When employing absorbable nails, one must avoid harsh hammering to prevent cortical penetration and subsequent implant failure.
The cartilage induced by the scaffold is hyaline-like cartilage, which is more similar to natural hyaline cartilage and has better performance than fibrocartilage	

MSC, mesenchymal stem cell.

## Disclosures

The authors declare the following financial interests/personal relationships which may be considered as potential competing interests: Y.A. is chairman of the International Cartilage Regeneration and Joint Preservation Society. Z.L. is a committee member of the International Cartilage Regeneration and Joint Preservation Society. All other authors (Z.H., T.P., W.Y., C.L., X.G.) declare that they have no known competing financial interests or personal relationships that could have appeared to influence the work reported in this paper.

## Funding

This work was supported by Beijing Natural Science Foundation - Haidian Original Innovation Joint Fund (L222065) (L242161), State General Administration of Sport Science and Technology Innovation Project (22KJCX007), Haidian Innovation Transformation Project (Scientific innovation Research and Development, HDCXZHKC2023201), Peking University Third Hospital talent incubation Fund (BYSYFY2021038) and Central government guided local science and technology development fund projects (246Z2004G).

## Supplementary Data

Video 1 The surgical technique of repairing cartilage injury with a tissue-engineering scaffold via a small incision. The first part is the arthroscopic image viewing from the anterior lateral portal of the patient’s left knee with the patient in the supine position. The second part is the direct viewing of the patient’s left knee with the patient in the supine position. First, establish the anteromedial (operation portal) and anterolateral (observation portal) portals to the knee joint. Then, explore and probe the defect area under arthroscopy. Clean the injured cartilage with a planer and the unstable cartilage at the edge of the injured area with a scraper. Use the microfracture device to perform the microfracture. After this, remove the arthroscope and expose the site of injury by a small incision. On the basis of the defect's size and location, create a minimally invasive incision of approximately 3 cm near the defect. Then, clean the injured cartilage with a scraper to stabilize the edge of the cartilage again. Later, select and trim the decalcified bone scaffold under direct vision. Place the scaffold into the joint and fix the decalcified bone scaffold in the defect area using the self-developed honeycomb positioning guide. Through the channels on the fixator's surface, use drill bit to drill through the cancellous and cortical bones, creating a bone tunnel and then implant the cartilage nails. After securing the scaffold in place, trim its surface with a planer to ensure it is flush with the defect area. At last, check the stability and complete the surgery.
